# Using directed acyclic graphs to determine whether multiple imputation or subsample-multiple imputation estimates of an exposure-outcome association are unbiased

**DOI:** 10.1093/aje/kwaf265

**Published:** 2025-11-25

**Authors:** Paul Madley-Dowd, Rachael A Hughes, Maya B Mathur, Jon Heron, Kate Tilling

**Affiliations:** MRC Integrative Epidemiology Unit at the University of Bristol, Bristol, United Kingdom; Population Health Sciences, Bristol Medical School, University of Bristol, Bristol, United Kingdom; NIHR Biomedical Research Centre, University of Bristol, Bristol, United Kingdom; Centre for Academic Mental Health, Population Health Sciences, Bristol Medical School, University of Bristol, Bristol, United Kingdom; MRC Integrative Epidemiology Unit at the University of Bristol, Bristol, United Kingdom; Population Health Sciences, Bristol Medical School, University of Bristol, Bristol, United Kingdom; Quantitative Sciences Unit, Department of Medicine, Stanford University, Stanford, CA, United States; MRC Integrative Epidemiology Unit at the University of Bristol, Bristol, United Kingdom; Population Health Sciences, Bristol Medical School, University of Bristol, Bristol, United Kingdom; Centre for Academic Mental Health, Population Health Sciences, Bristol Medical School, University of Bristol, Bristol, United Kingdom; MRC Integrative Epidemiology Unit at the University of Bristol, Bristol, United Kingdom; Population Health Sciences, Bristol Medical School, University of Bristol, Bristol, United Kingdom

**Keywords:** statistical methodology, missing data, selection bias, directed acyclic graphs

## Abstract

Missing data are a pervasive problem in epidemiology, with multiple imputation (MI) a commonly used analysis method. MI is valid when data are missing at random (MAR). However, definitions of MAR with multiple incomplete variables are not easily interpretable and descriptions of graphical model-based conditions are not accessible to applied researchers. Previous literature shows that MI may be valid in subsamples, even if not in the full dataset. Practical guidance on applying MI with multiple incomplete variables is lacking. We present an algorithm using directed acyclic graphs to determine when MI will estimate an exposure-outcome coefficient without bias. We extend the algorithm to assess whether MI in a subsample of the data, in which some variables are complete, and the remaining are imputed, will be valid and unbiased for the exposure-outcome coefficient. We apply the algorithm to several simple exemplars, and in a more complex real-life example highlight that only subsample-MI of the outcome would be valid. Our algorithm provides researchers with the tools to decide whether to use MI in practice when there are multiple incomplete variables. Further work could focus on the likely size and direction of biases and the impact of different missing data patterns.

## Introduction

Missing data is a common problem in epidemiological studies.^1-4^ Much of the work on how to analyze incomplete data has focused on only one variable having missing values, whereas often there are multiple incomplete variables. The impact of missingness depends on (1) the target parameter, (2) the analysis model, and (3) the missingness mechanisms. We focus on the case where the target parameter is the effect of an exposure on an outcome estimated using a regression model, conditional on a set of covariates, and some of the analysis model variables are incomplete. We provide a glossary (Table 1; extended version in Table S1) of technical terms used in this paper.

**Table 1:** Definitions of technical terms (extended version available in the Supplementary Material).

**Term (abbreviation)**	**Definition**
Analysis model	The model applied to the data to estimate the estimand (quantity) of interest. The variables in the analysis model are termed the analysis variables (includes outcome, exposure, covariates).
Auxiliary variable	A variable that is not in the analysis model but that is included as a predictor in the imputation model to recover information about the incomplete variable(s). For our purpose, auxiliary variables need not be complete.
Imputation model	A model used to predict and impute missing values for a given incomplete variable, as part of the multiple imputation procedure. When implementing multiple imputation for multiple incomplete variables using chained equations a separate imputation model is specified for each incomplete variable.
m-backdoor criterion	The m-backdoor criterion holds if any paths between missingness indicators and incomplete analysis variables are blocked conditional on all the modeled and complete auxiliary variables (ie, complete auxiliary variables included in the imputation model) and the complete analysis variables.^5^ Mathur and Shpitser show proofs of the soundness and completeness of the m-backdoor criterion for imputation in their appendices.^5^
Missing at random (MAR)	Several definitions have been developed for the concept of MAR and they do not perfectly overlap (see the work of Doretti, Geneletti and Stanghellini for a detailed explanation^6^). We list the definitions below in the order they were conceptualized and not in alphabetical order.
Rubin-MAR (equivalent to Realized-MAR)	As first defined by Rubin^7^^,^^8^: “The missing data are missing at random if for each possible value of [the parameters for the hypothesized missingness mechanism], the conditional probability of the observed pattern of missing data, given the missing data and the value of the observed data, is the same for all possible values of the missing data.” This was later elaborated on by Seaman et al.^9^ and described as “Realized-MAR.” Seaman highlighted that the definition is (1) “a statement only about the realized missingness pattern and realized observed data, not about missingness patterns or observed data that could have been realized but were not” and (2) “a statement about a hypothesized missingness model, rather than necessarily the true missingness process.”
Everywhere-MAR	As defined by Seaman et al.^9^: “[T]he hypothesized missingness model always assumes that, for any value of the data, the probability of any possible missingness pattern, given the values of the corresponding observed elements and missing elements of the data, does not depend on the values of the missing elements.”
z-MAR	A graph-based definition by Mathur and Shpitser^5^: The data are z-MAR if the m-backdoor criterion holds.
Missing completely at random (MCAR)	Using Rubin’s definition, the probability of the realized missingness pattern does not depend on observed or unobserved data.^8^
Missing not at random (MNAR)	Using Rubin’s definition, the probability of the realized missingness pattern depends on unobserved data even after conditioning on observed data.^8^ Often also defined as the data being neither MAR nor MCAR.
Missingness	Whether a variable $X$ is incomplete = partially observed. Missingness is represented, as a variable, by a response indicator ${R}_X$ which contains 1 second for those individuals where $X$ is observed and 0 where $X$ is missing.
Missingness pattern	The combination of responses/non-responses across all response indicators. For example, for two incomplete variables $X$ and $Y$, the possible missingness patterns are $\left({R}_X,{R}_Y\right)=\left(1,1\right)=$ both variables observed, $\left({R}_X,{R}_Y\right)=\left(1,0\right)=X$ observed and $Y$ missing, $\left({R}_X,{R}_Y\right)=\left(0,1\right)=X$ missing and $Y$ observed, and $\left({R}_X,{R}_Y\right)=\left(0,0\right)=X$ and $Y$ missing.
Subsample-multiple imputation (subsample-MI)	Restriction of the sample to only those participants with observed values for a subset of the incomplete variables and then (following restriction) application of multiple imputation to impute the remaining incomplete variables.^10^
Target parameter	The parameter of interest to the analyst. In our setting, using a regression model of the outcome on an exposure conditional on a set of covariates, the target parameter is the conditional regression coefficient for the exposure.

The simplest approach to analyzing incomplete data is to use complete records analysis (CRA) where individuals with missing data in any variable are excluded. The target parameter can be estimated without bias using CRA when the probability of inclusion in the analysis is independent of the outcome variable conditional on the covariates^3^^,^^11^. Directed acyclic graphs (DAGs)^12^^,^^13^ can be extended by including a response indicator for each incomplete variable (also called m-DAGs)^14^^,^^15^, and used to determine whether a target parameter can be estimated without bias by CRA.^3^^,^^16^ However, even where the estimate is unbiased using CRA, there may be loss of efficiency compared to the full-data estimate (ie, had there been no missing data), due to the reduced sample size.^17^

Multiple imputation (MI) is an alternative to CRA that may improve efficiency and may also estimate the target parameter without bias in some situations where estimation using CRA is biased.^7^^,^^18^ Imputation models must be correctly specified, compatible with the analysis model, include all analysis model variables and may also include auxiliary variables. The target parameter will be estimated without bias using MI if data are missing at random (MAR), conditional on the observed values of the variables included in the imputation model—ie, the probability of the realized missingness pattern does not depend on unobserved data, conditional on the realized, observed data.^7^^,^^8^ Guidelines for dealing with missing data, or for implementing MI, tend to emphasize the need to assess the plausibility of the MAR assumption—but give no details on how this might be done.^4^^,^^18-22^

**Figure 1:**
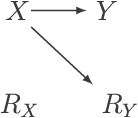
Directed acyclic graph (DAGs) where the variable $X$ is the exposure, $Y$ is the outcome and ${R}_{\mathrm{X}}$ and ${R}_{\mathrm{Y}}$ represent response indicator variables equal to 1 when $X$ and $Y$ are observed (ie, they are not missing) respectively. We do not include any boxes around variables to allow the DAGs to represent the data generating mechanism and not a specific estimator (such as complete records analysis or multiple imputation). The target parameter (the regression coefficient for the effect of *X* on *Y*) will be estimated without bias using complete records analysis and may be estimated with bias when using multiple imputation including all study participants.

The MAR assumption is usually presented in context of a single incomplete variable but is easily misunderstood or misapplied when there are multiple partially observed variables. Consider two partially observed variables, exposure $X$ and outcome $Y$, where missingness in $X$ does not depend on any other variable and missingness in $Y$ depends on $X$ (Figure 1, for expanded discussion see Supplementary Material). Among individuals with an observed $X$, missingness in $Y$ is independent of unobserved data given observed data ($X$). However, among individuals with a missing $X$, missingness in $Y$ depends on unobserved data ($X$), which contradicts the definition of MAR. Thus, the example in Figure 1 would be described as missing not at random (MNAR; ie, not MAR nor missing complete at random, MCAR).

The shortcomings of Rubin’s definition of MAR^8^ (referred to as “Rubin-MAR” from now on with “MAR” used to specify the assumption by any definition) have been explored elsewhere.^5^^,^^6^^,^^9^^,^^14^^,^^23-25^ Rubin-MAR refers only to the realized data and response indicators—that is, the values they take in the single dataset at hand. An alternative definition “everywhere-MAR”^9^ extends Rubin-MAR to all possible repeated samples of both the data and response indicators. Graphical approaches, based on DAGs^12^^,^^13^, have been developed^26^ and extended^5^ to enable researchers to assess whether data are MAR based on substantive knowledge and causal assumptions, though these definitions do not perfectly align with Rubin-MAR, nor everywhere-MAR.^6^ Using DAGs, Mathur and Shpitser defined the “m-backdoor criterion” which holds if “any paths between [response] indicators and incomplete analysis variables are blocked conditional on [all] the modelled [and complete] auxiliary variables and the complete analysis variables.”^5^ If the m-backdoor criterion holds then the data are “z-MAR.” As they formalize, the m-backdoor criterion (and therefore z-MAR) is sufficient for imputation to be valid for all distributions compatible with a given graph, and for recovering the joint distribution of all imputation model variables.^5^ This means we can unbiasedly estimate any parameter that can be derived using that joint distribution, including means and conditionally or marginally adjusted exposure-outcome associations. Using this criterion, we can see that the data in Figure 1 are not z-MAR as there is an open path from both $Y$ and $X$ to a response indicator, and so MI may not be valid. However, the descriptions of these graphical approaches are not easily accessible to applied researchers.

There is little accessible information on how to assess plausibility of MAR in practice, and little guidance in the epidemiological literature on how to proceed if MAR is not considered plausible, other than to use sensitivity analyses which are mostly framed in terms of “MNAR” as specifying differences in distributions between different missing data patterns. To handle certain forms of MNAR Little and Zhang have developed a “subsample-MI” approach, in which the dataset is restricted to a subsample of individuals with observed values for some incomplete variables, and the MI procedure is applied to the remaining incomplete variables in this subsample only.^10^ The estimated target parameter may be unbiased using subsample-MI even when it is biased using MI of the whole dataset. Little and Zhang show that the conditional exposure-outcome association (often estimated as a regression coefficient), is unbiased using subsample-MI when:

1. The probability of inclusion in the subsample does not depend on the outcome variable conditional on the covariates in the analysis model (including the exposure).
2. Within the subsample, the data are MAR (the authors used Rubin-MAR).

Intuitively, the first condition ensures that the subsample estimate of the conditional exposure-outcome association (our target parameter) is an unbiased estimate of the full-data-parameter.^3^^,^^14^ The second condition ensures that MI is valid within the subsample; this would also apply to other target parameters (eg, the mean of the outcome).

The purpose of the current paper is to bridge the divide between applied researchers and methodological foundations for validity of MI, by providing an algorithm, and showing how to operationalize it using DAGs, to (1) assess whether MI applied to the whole dataset can estimate the exposure-outcome association without bias using a regression model and, if not, (2) identify whether the association can be estimated without bias using MI applied to a subsample of the data.

## Motivating example

We demonstrate the practical issues using a previously described example^27^ from the Avon Longitudinal Study of Parents and Children.^28-30^ The analysis model was a linear regression of offspring intelligence quotient (IQ) scores at age 15 years (the outcome) on maternal smoking during pregnancy (the exposure), conditionally adjusted for maternal age, education and parity (we describe these variables collectively as proxies for socioeconomic position; SEP),^31-35^ and offspring sex. The proxies for SEP are probable confounders; offspring sex is not a confounder but may explain some of the variation in IQ.^36^ Linked education score at age 16 years was available as an auxiliary variable to impute IQ at age 15 years. The exposure, outcome, proxies for SEP, and auxiliary variable were all incomplete, while offspring sex was complete.

**Figure 2:**
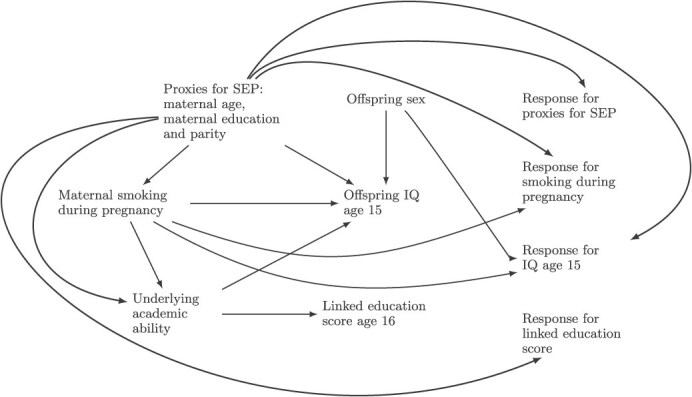
Directed acyclic graph of the assumed relationships between variables and response indicators in an applied example from the Avon Longitudinal Study of Parents and Children investigating the effect of maternal smoking during pregnancy (exposure) on offspring intelligence quotient (IQ) scores at age 15 years (outcome). In this example the analysis model is a linear regression of the outcome on the exposure adjusted for proxies for socioeconomic position (SEP) and offspring sex. Linked education score at age 16 years is being used as an auxiliary variable for the incomplete outcome. Underlying academic ability is an unmeasured variable.

Figure 2 shows a (simplified) DAG of the assumed relationship between variables and response indicators. Informed by prior research,^37^ we include arrows from proxies for SEP to all response indicators, from maternal smoking during pregnancy to its own response indicator and to that for offspring IQ, and from offspring sex to the response indicator for offspring IQ. Applying the m-backdoor criterion to the DAG,^5^ we can see that the data are not z-MAR as there are open paths between incomplete variables and response indicators that are not blocked by conditioning on complete analysis model variables or complete auxiliary variables. Therefore, MI may not be valid for estimating the conditional exposure-outcome association without bias. However, applied researchers not familiar with literature on m-DAGs may find it challenging to arrive at this conclusion. Guidance so far has not incorporated the exploration of whether the z-MAR assumption may hold in a subsample.

## Establishing the validity of multiple imputation and subsample-multiple imputation for a specified DAG

In this paper, the target parameter is the coefficient for exposure $X$ from a regression of outcome $Y$ on $X$ and covariates $W$. Auxiliary variables $A$ (which are not in the analysis model) may be included in the imputation model(s). Any of these variables $\left\{\mathrm{Y},\mathrm{X},\mathrm{W},\mathrm{A}\right\}$ may be incomplete, with response indicator ${R}_J$ for variable $J$, where ${R}_{Ji}=1$ if variable $J$ is observed for individual $i$, and 0 otherwise. We define any variables ${V}_1$ and ${V}_2$ as *dependent* conditional on ${V}_3$ if the underlying variables ${V}_1$ and ${V}_2$ (not the observed, potentially incomplete, variables) are connected by an open path (known as d-connected) when we condition on ${V}_3$. In other words, “${V}_1$ and ${V}_2$ are dependent” does not mandate a direct causal path between ${V}_1$ and ${V}_2$.^12^^,^^13^ We assume for simplicity that the researcher chooses auxiliary variables a priori and includes the same set of variables throughout the algorithm's implementation. Note that the algorithm does not search for possible sets of auxiliary variables; rather, the algorithm is used to assess whether imputation is valid using the chosen set. If a different set of auxiliary variables is chosen later, eg, if the analyst obtains additional sources of data, the algorithm must be repeated.

Our primary aim is to establish whether MI applied to the full dataset (ie, imputing all incomplete variables and for the whole sample) can estimate the target parameter without bias, by establishing whether the m-backdoor criterion holds.^5^ We assume throughout that the MI is carried out correctly, including that all imputation models are correctly specified, and all imputation models are compatible with the analysis model.^21^ We use some extensions to standard DAG conventions (summarized in Table 2) to allow complete/incomplete variables to be easily distinguished:

**Table 2:** Guide to the interpretation of additional DAG notation implemented within the algorithm.

**Suggested additional DAG notation**	**Meaning**
Dashed red boxes	Complete measured variables
Dashed green circles	Incomplete or unmeasured variables
Dotted blue circles	Incomplete measured variables with an open path to any response indicator

Note that the colors and line types are suggestions and can be adapted to make the algorithm more accessible (eg, for color blind users) or for use in black and white print. However, we suggest implementing our shapes (in particular, using a box for complete variables) as this highlights via conventional DAG notation that conditioning on complete variables (only) can be used to block open paths between incomplete variables and response indicators.

**Step 1:** Draw the DAG showing the assumed causal relationships between all analysis model variables $\left\{Y,X,W\right\}$, any auxiliary variables $A$, and the set of response indicators $R$ for each incomplete variable in $\left\{Y,X,W,A\right\}.$^14^ Include unmeasured variables $U$ as appropriate (eg, unmeasured common causes of $\left\{Y,X,W,A,R\right\}$).^38^ We let $Z$ be the set of complete variables, and ${Z}^{\prime }$ the set of incomplete variables. Indicate which variables are complete variables $Z$ (eg, using a red dashed box), and which are incomplete variables ${Z}^{\prime }$ (eg, using a green dashed circle). Unmeasured variables $U$ are considered incomplete (ie, included in ${Z}^{\prime }$), but do not have a response indicator, cannot be conditioned on, and will never be imputed.

**Step 2:** Identify all measured incomplete variables that have an open path to *any* response indicator in $R$, conditional on complete variables $Z$ (ie, conditioning only on those variables with a red dashed box). Indicate this set as $\Phi$, using for example a blue dotted circle around all such variables in the DAG.

**Step 3:** Check whether the m-backdoor criterion holds.

- If $\Phi$ is empty (ie, no variables have a blue dotted circle around them), then the m-backdoor criterion is met, the data are z-MAR, and MI estimates will be unbiased.
- If $\Phi$ is not empty (one or more variables have a blue dotted circle around them) then the m-backdoor criterion is not met, the data are not z-MAR, and MI estimates may not be valid.

We note in step 3 that MI estimates “may not,” as opposed to “will not” be valid as there may be situations where the data are not z-MAR but MI estimates will still be valid. Examples include: (1) specific realized missingness patterns (eg, in Figure 1, if no participant has both $X$ and $Y$ missing at the same time then the data are Rubin-MAR but not z-MAR^9^); and (2) specific parameterizations, such as estimating the target parameter using logistic regression, when MI of the outcome is valid when only the outcome causes its own missingness.^3^

If the m-backdoor criterion does not hold, then estimates using MI applied to the whole dataset may not be valid. We can then explore whether our target parameter can be unbiasedly estimated via subsample-MI by repeating the algorithm, using the following modifications to step 1:

**Step 1, modification 1:** Draw the DAG as before, comprised of $\left\{Y,X,W,A,U,R\right\}$. Partition the measured incomplete variables into subsets *P* and *Q*, where *P* contains variables that will be imputed within the subsample of individuals with complete data on variables in *Q*. The subset *Q* must contain only variables with a response indicator that is independent of *Y* conditional on analysis model variables 𝑋 and 𝑊 (which may be complete or incomplete; note this is different to the m-backdoor criterion). Multiple options for subsets *P* and *Q* may be possible and would need to be explored separately. *U* are excluded from both *P* and *Q* but auxiliary variables *A* may be included in either set (as appropriate).

**Step 1, modification 2:** Condition on all response indicators for variables in $Q$ (draw boxes around them to indicate this, as usual convention). We recommend also indicating that these response indicators are set to 1 in this subsample. Next, add red dashed boxes to all variables in $Z$ and to all variables in $Q$ (as these are “complete” in the subsample defined above). Add green dashed circles to all variables in $P$.

Proceed with steps 2 and 3 of the algorithm using the new DAG for the subsample in which variables in $Q$ are complete. If in step 3 there are no open paths between incomplete variables in $P$ and response indicators for variables in $P$, then the m-backdoor criterion holds in the subsample and MI of the variables in $P$, applied only to the subsample of individuals with complete $Q$, will be unbiased for the target parameter. If there are such open paths, then the m-backdoor criterion does not hold in the subsample, and MI of the variables in $P$, applied only to the subsample of individuals with complete $Q$, may be biased. In such a situation, alternative approaches could include considering different subsamples (defined by other choices of sets $P$ and $Q$) or identifying further auxiliary variables to block paths between the incomplete variables and response indicators and repeating the entire algorithm.

### Worked examples of implementation of the algorithm

**Figure 3:**
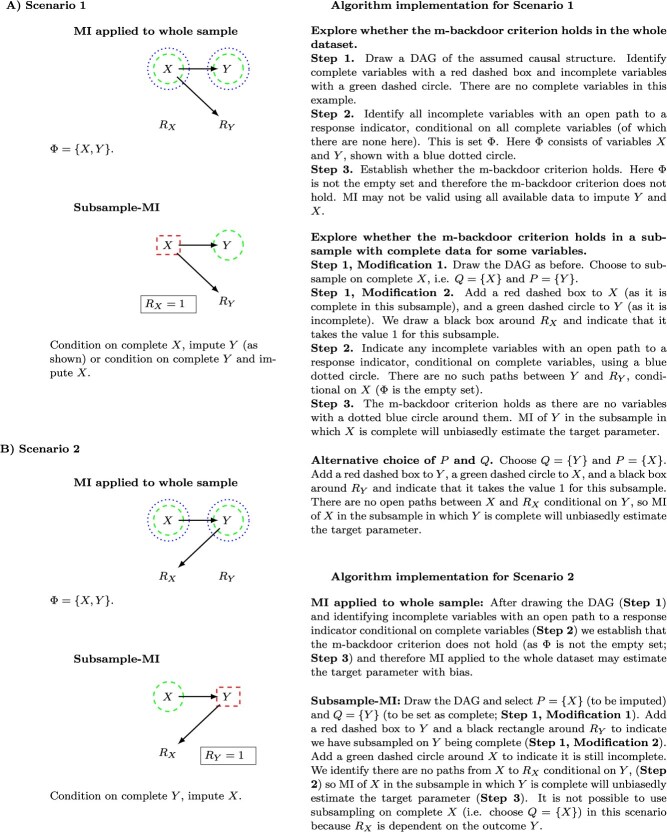
Examples of the algorithm’s implementation being applied to the directed acyclic graphs presented in Figure 1 and Figure S1. The target parameter is the regression coefficient for X in a regression of Y on X.

**Figure 4:**
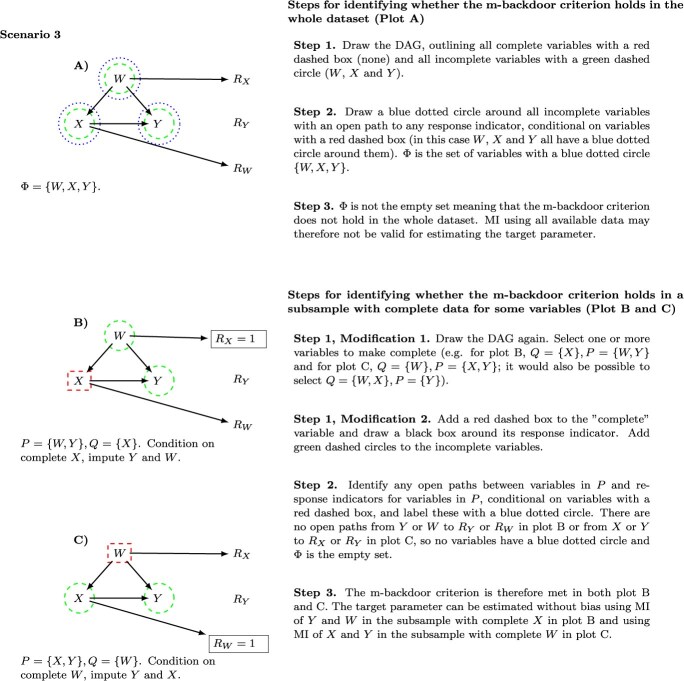
Worked example of algorithm implementation involving three variables where W is a confounder of X and Y, with W causing missingness in X, X causing missingness in W, and missingness in Y not caused by any variable. The target parameter is the regression coefficient for X in a regression of Y on X and W.

We apply the algorithm to three scenarios (Figures 3A, B and 4; see Supplementary Material for an accompanying simulation study comparing bias in estimates made using CRA, MI applied to the whole sample, and subsample-MI).

In scenario 1 (Figure 3A; the same DAG as Figure 1), $X$ causes $Y$ and ${R}_Y$, there are no causes of ${R}_X$. In steps 1 to 3, we show that $\Phi$ consists of variables $X$ and $Y$ and therefore that MI will not be valid using all available data to impute $Y$ and $X$ (because $\Phi$ is not the empty set). We next examine whether there are any subsamples in which use of MI would enable us to estimate the target parameter without bias.

We could investigate imputing $Y$ in the subsample in which $X$ is complete, or vice-versa. $Y$ is not associated with the response indicator for either $X$ or $Y$, conditional on $X$, so either choice is admissible. Following the modifications to step 1 and then applying steps 2 and 3, the algorithm indicates that MI restricted to a subsample of either complete $X$ ($Q=\left\{X\right\}$ and $P=\left\{Y\right\}$) or complete $Y$($Q=\left\{Y\right\},P=\left\{X\right\}$) will be valid. Thus, here, we have a choice of strategies. The analyst could carry out both options and compare the results, or might favor the subsample with the largest sample size. Imputing only the outcome without any auxiliary information will not be more statistically efficient than CRA (because the imputation model and the analysis model are the same, and MI uses no extra information) so in this example we prefer to use $Q=\left\{Y\right\}$ and $P=\left\{X\right\}$ (further explanation provided in box 2 of Hughes et al.^3^).

In scenario 2 (Figure 3B; the same DAG as in Figure 1 but with a different missingness mechanism—see also Figure S1 in the supplement), $X$ causes $Y$, $Y$ causes ${R}_X$ and there are no causes of ${R}_Y$. Following the algorithm, we conclude that MI in the full sample may not be valid, but MI of $X$ applied to the subsample in which $Y$ is fully observed will be valid (MI of $Y$ in the subsample with complete $X$ may not be valid as $Y$ causes ${R}_X$).

In scenario 3 (Figure 4A-C), $W$ is a confounder of $X$ and $Y$, with $W$ causing missingness in $X$, $X$ causing missingness in $W$, and missingness in $Y$ being independent of all other variables. Steps 1-3 determine that $\Phi$ contains $W$, $X$ and $Y$, and therefore MI using all available data may not be valid (because $\Phi$ is not the empty set).

We now need to decide how to separate the measured incomplete variables in ${\mathrm{Z}}^{\prime }$ into sets $P$ and $Q$. As no response indicators are dependent on the outcome given analysis model covariates $X$ and $W$, we can explore multiple possible sets $P$ and $Q$. We could choose variable $X$ as the “complete” variable in modification 1 of step 1 (Figure 4B), ie, $Q$ is “complete” ($X$) and $P$ is “to be imputed” ($Y$ and $W$). Using the algorithm, we identify that there is no open path from $Y$ or $W$ to ${R}_Y$ or ${R}_W$ and thus the subsample-MI, where $Y$ and $W$ are imputed in the subsample with complete $X$, is valid.

Similar logic shows that if we instead choose to subsample on complete $W$ (Figure 4C) then MI of $Y$ and $X$ in this subsample will estimate the target parameter without bias. MI in the subsample with $Y$ complete is not valid as the data are not z-MAR within the subsample (there are open paths between incomplete variables $W$ and $X$ and response indicators). The target parameter could also be estimated without bias by subsampling on complete $X$ and $W$, and imputing $Y$, though this would be less statistically efficient than choosing either $X$ or $W$ as the complete variable.

### Further examples

The Supplementary Material includes further examples that explore (1) subsampling on an incomplete outcome variable (Figure S2A), (2) subsampling in the presence of an unmeasured variable (Figure S2B), (3) subsampling on multiple incomplete variables (Figure S2C), and (4) subsampling with an incomplete auxiliary variable (Figure S3). The first three of these examples are explored in the supplementary simulation study. We additionally apply the algorithm to the canonical DAGs presented by Moreno-Betancur et al.^14^^,^^15^

### Application to the motivating example

We apply the algorithm to the motivating example (Figure 2) (see Supplementary Material  Figures S4 and S5 showing implementation of the algorithm). Following steps 1 to 3, we identify the set $\Phi$ = {maternal smoking during pregnancy, IQ at age 15 years, proxies for SEP, linked education score}. As $\Phi$ is not the empty set, the m-backdoor criterion is not met and MI applied to the whole dataset may not be valid. We set $Q$ = {maternal smoking during pregnancy, proxies for SEP} and $P$ = {IQ at age 15 years, linked education score}. As there are no open paths from the set $P$ to response indicators for variables in $P$, MI of IQ at age 15 years and linked education score in the sample with complete proxies for SEP and maternal smoking during pregnancy will be valid.

The conclusions from the algorithm require the DAG to be true. In this case, there may be additional arrows not included in Figure 2 (see discussion of our previous work^27^): it is likely that the response indicators for IQ at age 15 years and linked education score are caused by IQ at age 15 years and linked education score, respectively. Applying the algorithm to the updated DAG incorporating these relationships would indicate that neither MI applied to the full dataset, nor MI applied to any subsample, would be valid. Examples of assessing the m-backdoor criterion when the DAG is only partially known are provided by Mathur and Shpitser and could be used to aid decision making.^5^

## Discussion

We have built on previous work in DAGs^3^^,^^14^^,^^15^, z-MAR and the m-backdoor criterion,^5^ and subsample-MI,^10^ to provide guidance for deciding how (or whether) to apply MI to estimate an exposure-outcome association using a regression model. This fills a crucial gap in the advice to applied researchers on how to deal with multiple incomplete variables when implementing MI.^4^ The key point is that MI is valid if all incomplete variables are independent of all response indicators, conditional on the complete variables. Identifying a subsample within which all incomplete variables are independent of response indicators, given complete variables, can lead to unbiased estimates of the conditional exposure–outcome association, even where neither CRA, nor MI on the full sample, would do so.

Critically, there are additional considerations when it comes to outcome $Y$. If $Y$ cannot be d-separated from its own response indicator by the complete variables—for example, because $Y$ is the direct cause of its own missingness, or if there is an unmeasured common cause of $Y$ and missingness in $Y$—then typically both CRA and MI (in any subsample of the data) will yield biased estimates of the regression coefficient for the effect of $X$ on $Y$. There are well-known exceptions for certain models, though, such as logistic regression. Thus, one preliminary step before commencing the algorithm could be to examine the DAG and assess whether $Y$ is d-connected to its response indicator either directly or via an unmeasured common cause. However, some other scenarios where $Y$ is d-connected to its own response indicator could lead to unbiased estimates in subsamples—for example, if incomplete $X$ causes missingness in $Y$ (as in Figures 1 and 3A).

The relation between response indicators for incomplete variables and $Y$ is a key consideration for deciding which variables to use in subsampling and which to impute (division into sets $P$ and $Q$). The analyst must check (1) whether the response indicator for each incomplete variable is d-connected to $Y$ given analysis model variables, as such d-connected variables cannot be used for subsampling (and can therefore not be in set $Q$) and (2) whether an incomplete variable is related to its own response indicator (either as a direct cause or via an unmeasured variable) as such variables cannot be imputed (and can therefore not be in set $P$). If any variable cannot either be used for subsampling or be imputed (ie, cannot be in either set $P$ or set $Q$), then valid MI cannot be conducted in any subsample.

This algorithm will be most useful when there are multiple variables with missing data, and arbitrary missing data patterns. This commonly occurs in the analysis of data from cohort studies, especially where data from different waves of data collection are used. Often it is plausible that baseline covariates (such as measures of economic hardship) may affect likelihood of responding at any wave.^37^ If these measures are themselves incomplete (so the data potentially not z-MAR), then MI restricted to those with complete data on economic hardship may be unbiased. Linkage of cohorts to other, more complete sources (eg, electronic healthcare records) means that restricting MI to samples with complete outcome data may be useful.^39^ In RCTs, baseline covariates are usually complete, and the concern is about missing outcome data. Intermediate measures of the outcome may be used to impute the final outcome—either overall, or separately within each arm of the trial.^40^ If these variables are also incomplete, then again, our algorithm may be useful to decide on the best analysis strategy.

As with any other methodology based on DAGs (such as confounder selection, or assessment of plausibility of bias), the conclusions depend critically on the DAG assumed. These assumptions should be justified based on external knowledge or prior information (such as documented reasons for missingness). Temporality may help to eliminate some paths—for example, if $Y$ is measured at 12-month follow-up then there cannot be a direct arrow from $Y$ to the response indicator for a variable measured at baseline. Tests of whether the DAGs are incompatible with the dataset can be used,^41^ and sensitivity analyses conducted to examine robustness to assumptions about which there is uncertainty.^16^ As recommended by Moreno-Betancur et al, conclusions should be examined across a range of plausible m-DAGs.^14^^,^^15^

### Limitations and further work

There are some limitations to our work. MI is not the only method for dealing with incomplete data—others include inverse probability weighting and maximum likelihood methods, which we have not examined here.^42^ For MI to unbiasedly estimate the target parameter depends on more than just the conditions outlined here—we have assumed throughout that all imputation and analysis models are correctly specified and are compatible with the analysis model.^43^ A limitation of the use of DAGs is that they give no information about the magnitude or direction of any bias. As noted earlier, the algorithm is conservative and in certain circumstances may conclude that MI or subsample-MI is biased when no bias exists.

Future work could focus on considerations when selecting the subsample to use for MI (ie, the selection of variables in $Q$)—these could include the amount of missingness in each variable (as this will affect the sample size), and the fraction of missing information (as this will affect the efficiency of MI). Automation of the identification of possible sets $P$ and $Q$ based on a user-specified DAG is underway and will be incorporated into the *midoc* R package.^44^ We have noted that imputing just the outcome using only analysis model variables will not aid in the precision of the target parameter estimate,^3^ which suggests that some subsample selections will be more useful than others. We have assumed for convenience that the same auxiliary variables are included in all imputation models, though this may not be necessary. Further work is needed to aid the identification of appropriate and necessary auxiliaries in the context of subsample-MI.

## Conclusion

We have provided an easy-to-implement algorithm to enable researchers to decide how plausible it is that MI applied to the full dataset will estimate a target exposure–outcome association without bias, for a given assumed causal structure. Application of a previously derived concept of “subsample-MI”^10^ provides a rationale for exploring subsamples in which MI is valid, even when MI in the whole dataset is not.

## Supplementary Material

Web_Material_kwaf265

## Data Availability

Code for the simulations can be found at https://github.com/pmadleydowd/Subsample-MI.
