# Supplementary material for: Using directed acyclic graphs to determine whether multiple imputation or subsample-multiple imputation estimates of an exposure-outcome association are unbiased
Source: Am J Epidemiol. 2025 Nov 25;195(2):505–14. doi: 10.1093/aje/kwaf265 (PMC13368849; doi:10.1093/aje/kwaf265)
Supplement: Web_Material_kwaf265 [file web_material_kwaf265.docx]

Supplementary materials

Using directed acyclic graphs to determine whether multiple imputation or subsample-multiple imputation estimates of an exposure-outcome association are unbiased

Contents

[Supplementary tables 2](#_Toc211956332)

[Table S1. Extended definitions of terms and abbreviations 2](#_Toc211956333)

[Extended background 5](#_Toc211956334)

[Extended further examples 6](#_Toc211956335)

[Algorithm applied to canonical missingness DAGs of Moreno-Betancur et al 2018 ^8^ 9](#_Toc211956336)

[Figures showing application of algorithm to the motivating example 18](#_Toc211956337)

[Simulation study 20](#_Toc211956338)

[Simulation study methods 20](#_Toc211956339)

[Aims 20](#_Toc211956340)

[Data-generating mechanisms 20](#_Toc211956341)

[Estimand/target of analysis 23](#_Toc211956342)

[Methods to be evaluated 23](#_Toc211956343)

[Performance measures 23](#_Toc211956344)

[Simulation study results 24](#_Toc211956345)

[Supplementary references 25](#_Toc211956346)

# Supplementary tables

## Table S1. Extended definitions of terms and abbreviations

| **Term (abbreviation)** | **Definition** |
| --- | --- |
| Analysis model | The model applied to the data to estimate the estimand (quantity) of interest. The variables in the analysis model are termed the analysis variables (includes outcome, exposure, covariates). |
| Auxiliary variable | A variable that is not in the analysis model but that is included as a predictor in the imputation model to recover information about the incomplete variable(s). For our purpose, auxiliary variables need not be complete. |
| Compatibility of the analysis and imputation models | Zahid, Faisal and Heumann state that “Two conditional densities are compatible if a joint distribution exists that has the given densities as its conditional densities”^1^. In the context of multiple imputation, the analysis model and imputation models are compatible if they can be derived as conditional models from a joint model. Practically, one implication of this is that they must at the very least contain the same variables in the same form, including any interaction terms^2^. |
| Complete records analysis (CRA) | Estimation of the target parameter using an analysis model that excludes any observation with unobserved (missing) values in any analysis model variable. This has often been referred to as complete case analysis in the literature, which we avoid using due to the potential for confusion with cases of a disease. |
| Collider variable | A variable that is caused by two other variables. Conditioning on a collider variable, for example via conditional adjustment or by selecting the sample for which this variable takes a particular value, may induce an association between the two “parent” variables even if, unconditionally, the two variables are independent^3^. |
| Correct specification of the analysis model and each imputation model | The analysis model and each imputation model include all the relevant variables (e.g. those necessary to account for confounding, selection and information bias ^4^), as well as any interaction terms, and all variables have the correct functional form^2^. |
| Directed acyclic graphs (DAGs) | Graphs consisting of nodes (representing variables) and directed edges (representing causal direction) used to represent the assumed causal relationship between variables^5 6^. |
| d-connected | Two variables are d-connected if there is an open path between them (see path definition below)^5-7^. |
| d-separated | Two variables are d-separated if there is no open path between them (see path definition below)^5-7^. |
| m-DAG | An extension of DAGs that additionally includes a missingness/response indicator for each incomplete (partially observed) variable^8 9^. |
| Fraction of missing information (FMI) | A parameter-specific measure of the amount of information lost due to missing data under a given analysis model and imputation model. This can be conceptualised as the fraction of the total variance in MI estimates that is attributable to the between imputation variance, though Carpenter and Kenward show a more accurate estimate of the FMI that is used in most software packages (p. 40)^10^. In practice, the FMI is often obtained by running the multiple imputation procedure and requires a large number of imputations to be estimated reliably. |
| Imputation model | A model used to predict and impute missing values for a given incomplete variable, as part of the multiple imputation procedure. When implementing multiple imputation for multiple incomplete variables using chained equations a separate imputation model is specified for each incomplete variable. |
| m-backdoor criterion | The m-backdoor criterion holds if any paths between missingness indicators and incomplete analysis variables are blocked conditional on all the modelled and complete auxiliary variables (i.e. complete auxiliary variables included in the imputation model) and the complete analysis variables^11^. Mathur and Shpitser show proofs of the soundness and completeness of the m-backdoor criterion for imputation in their appendices^11^. |
| Missing at random (MAR) | Several definitions have been developed for the concept of MAR and they do not perfectly overlap (see the work of Doretti, Geneletti and Stanghellini for a detailed explanation^12^). We list the definitions below in the order they were conceptualised and not in alphabetical order. |
| Rubin-MAR (equivalent to Realized-MAR) | As first defined by Rubin^13 14^: “The missing data are missing at random if for each possible value of [the parameters for the hypothesized missingness mechanism], the conditional probability of the observed pattern of missing data, given the missing data and the value of the observed data, is the same for all possible values of the missing data”. This was later elaborated on by Seaman et al.^15^ and described as “Realized-MAR”. Seaman highlighted that the definition is 1) “a statement only about the realised missingness pattern and realised observed data, not about missingness patterns or observed data that could have been realised but were not” and 2) “a statement about a hypothesised missingness model, rather than necessarily the true missingness process”. |
| Everywhere-MAR | As defined by Seaman et al.^15^: “[T]he hypothesised missingness model always assumes that, for any value of the data, the probability of any possible missingness pattern, given the values of the corresponding observed elements and missing elements of the data, does not depend on the values of the missing elements.” |
| v-MAR | A graph based definition by Mohan and Pearl^16^: “In graphical terms, v-MAR holds if (i) no edges exist between [a response indicator] and any partially observed variable and (ii) no bidirected edge exists between [a response indicator] and a fully observed variable.” |
| z-MAR | A graph-based definition by Mathur and Shpitser^11^: The data are z-MAR if the m-backdoor criterion holds. The definitions of v-MAR and z-MAR are related: v-MAR is a special case of z-MAR with no auxiliary variables. |
| Missing completely at random (MCAR) | Using Rubin’s definition, the probability of the realized missingness pattern does not depend on observed or unobserved data^13^. |
| Missing not at random (MNAR) | Using Rubin’s definition, the probability of the realized missingness pattern depends on unobserved data even after conditioning on observed data^13^. Often also defined as the data being neither MAR nor MCAR. |
| Missingness | Whether a variable $X$ is incomplete = partially observed. Missingness is represented, as a variable, by a response indicator $R_{X}$ which contains 1s for those individuals where $X$ is observed and 0 where $X$ is missing. |
| Missingness mechanism/Missingness model | The process by which a variable is probabilistically observed or not. Represented in an m-DAG by the variables that cause the response indicator for a given variable. |
| Missingness pattern | The combination of responses/non-responses across all response indicators. For example, for two incomplete variables $X$ and $Y$, the possible missingness patterns are $\left( R_{X},R_{Y} \right)=\left( 1,1 \right) =$ both variables observed, $\left( R_{X},R_{Y} \right)=\left( 1,0 \right)=X$ observed and $Y$ missing, $\left( R_{X},R_{Y} \right)=\left( 0,1 \right)=X$ missing and $Y$ observed, and $\left( R_{X},R_{Y} \right)=\left( 0,0 \right)=X$ and $Y$ missing. |
| Multiple imputation (MI) | A modelling approach to handle missing data in which multiple datasets are created, in each of which missing values are imputed based on predictive models (the imputation models). The analysis model is then fitted in each imputed dataset and a combined effect estimate is obtained using Rubin’s rules^14^. |
| Path | As defined by Pearl, a path from node/variable A to node/variable B “any consecutive sequence of edges, disregarding their directionalities”^5 7^ that starts at A and ends at B. A path can feature any node (variable) only once. A “directed path” is a path that follows the directionality of edges. A path can be blocked either by 1) a pair of arrows that collide head-to-head at an unconditioned variable/node (known as a collider variable – further explanation above) or by 2) a conditioned-on variable/node that does not have a pair of arrows pointing into it (i.e. a non-collider variable) ^5 7^. |
| Closed path | A closed path is a path from A to B is one that is blocked according to the two rules above. |
| Open path | An open path (also referred to as an unblocked path) is a path that is not blocked according to the two rules above (i.e. it can be followed without going through either 1) an unconditioned collider variable/node or 2) a conditioned on non-collider variable/node)^5 7^. |
| Realized | The concepts of realized data and realized missingness are related to the sampling of the data from the target population and the sampling of the observed data from the realized data. Seaman et al. note the importance of not confusing the realized and observed values^15^. |
| Realized data | The realized data is the specific draw of the joint distribution of the data from the target population^15^. |
| Realized missingness | The realized missingness is the particular matrix of response indicators that arises in a given dataset^15^. |
| Observed data | The observed data is the realized missingness imposed on the realized data^15^. A fresh draw of the response indicators will result in a new realised missingness, and hence likely new observed data, but not new realized data. |
| Recoverability | Whether it is possible to consistently estimate a model parameter or target estimand from the available data^8 16 17^. |
| Response indicator | The indicator variable for a participant having observed data (i.e. equal to 1 if the participant responded and 0 if they did not respond) for a given variable. This is sometimes referred to as a missingness indicator in which case the indicator is equal to 1 if the participant did not respond and equal to 0 if they did respond (though there is little consistency in terms of notation in the literature). |
| Subsample-multiple imputation (subsample-MI) | Restriction of the sample to only those participants with observed values for a subset of the incomplete variables and then (following restriction) application of multiple imputation to impute the remaining incomplete variables^18^. |
| Target parameter | The parameter of interest to the analyst. In our setting, using a regression model of the outcome on an exposure conditional on a set of covariates, the target parameter is the conditional regression coefficient for the exposure. |

# Extended background

Consider two scenarios, presented in Figure 1 of the main text and in Figure S1 below, that were inspired by an example provided in an online blog by Paul Allison ^19^ and have been explored by Mohan and Pearl previously (see Figure 4 of their paper) ^16^. In both scenarios there are two variables, an exposure variable $X$ that causes an outcome variable $Y$, both of which are partially observed with response indicators $R_{X}$ and $R_{Y}$ equal to 1 when that variable is observed. Note that we have drawn Figure 1 and Figure S1 to represent the data generating mechanism only and so no boxes, representing conditioning, are included. To use the DAG to explore bias in a CRA, in accordance with DAG convention, boxes would be drawn around variables which are to be conditioned on in the analysis – in this case, only the exposure variable $X$. In the presence of incomplete data, a CRA also conditions on the response indicators, by restricting the analysis to the sample in which $X$ and $Y$ are observed (i.e. $R_{X}$ and $R_{Y}$ are both equal to 1, indicated by a box drawn around these response indicators). In the first scenario (Figure 1) missingness in $Y$ is caused by $X$ (indicated by the arrow from $X$ to $R_{Y}$), while in the second (Figure S1) missingness in $X$ is caused by $Y$. Based on the DAG it is easy to establish that the regression coefficient for the exposure-outcome effect will be unbiased using CRA for the first scenario as missingness does not depend on the outcome (Figure 1). In the second scenario (Figure S1) the estimate for the exposure-outcome effect may be biased using CRA, because missingness in $X$ is dependent on the outcome variable. In both scenarios the data are not MAR (nor z-MAR as the m-backdoor criterion is not met), therefore the estimate of the exposure-outcome coefficient may be biased using MI. This may be easy to see for these simple examples, as the missingness indicators are dependent on incomplete variables. However, in complex scenarios, with more incomplete analysis model variables, visually identifying such dependencies becomes more difficult.


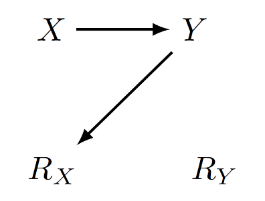


Figure S1: Directed acyclic graph (DAG) where the variable $X$ is the exposure, $Y$ is the outcome and $R_{X}$ and $R_{Y}$ represent response indicator variables equal to 1 when $X$ and $Y$ are observed (i.e., they are not missing) respectively. We do not include any boxes around variables to allow the DAGs to represent the data generating mechanism and not a specific estimator (such as complete records analysis or multiple imputation). The target parameter (the regression coefficient for the effect of X on Y) will be estimated with bias as missingness is dependent on the outcome and may be estimated with bias when using multiple imputation including all study participants. Figure S1 is the same as Figure 1 in the main text but with a different missingness mechanism.

# Extended further examples

Figure S2 shows the DAGs for further examples that explore A) subsampling on an incomplete outcome variable, B) subsampling in the presence of an unmeasured variable, C) the need in some circumstances to subsample on multiple incomplete variables. These examples are also explored in the simulation study later in the Supplementary material. Figure S2A-S2C use the same analysis model variables as the example presented in Figure 4 of the main text but have different missingness mechanisms.


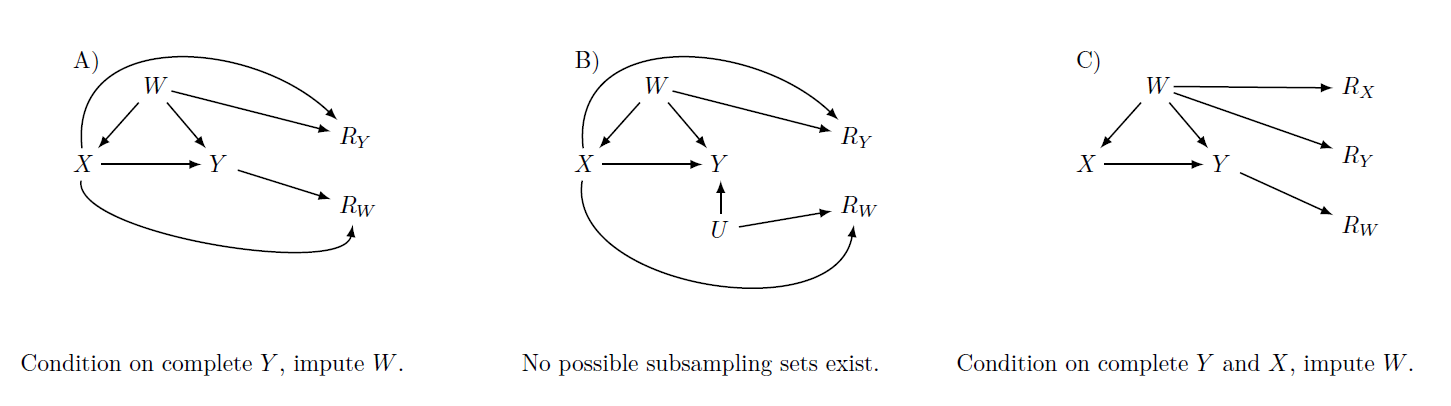


Figure S2: Directed acyclic graphs for the further examples involving three analysis model variables. In each the target parameter is the regression coefficient for X in a regression of Y on X and W.

In Figure S2A the confounder $W$ and exposure $X$ cause $R_{Y}$, both $Y$ and $X$ cause $R_{W}$, and $X$ is complete. Following steps 1-3, the set $\Phi$ consists of $W,Y$. As this is not the empty set MI applied to the whole dataset may not be valid. The set of incomplete variables, $Z’$, also consists of $\{W,Y\}$. Separating this set into $P$, incomplete variables whose response indicator is dependent on $Y$ conditional on analysis model variables, and $Q$, incomplete variables whose response indicator is independent of Y conditional on analysis model variables, gives $P=W$ and $Q=Y$. We restrict the sample to observed values for all variables in $Q$ (i.e. restrict to observations with observed $Y$), and check whether the variables in $P$ (i.e. $W$) are independent of all remaining response indicators (i.e. response indicators for variables in $P$, here just $R_{W}$) conditional on all complete variables ($Z=X$), all variables in $Q$ (i.e. $Y$), and all response indicators for variables in $Q$ (i.e. $R_{Y}=1$). Restriction to observed $Y$ eliminates the path from $W$ to $R_{Y}$ in the subsample and there is no open path from W to $R_{W}$ conditional on $X$ and $Y$ (which would be included in the imputation model for $W$), meaning that this check is passed. The data are therefore z-MAR in the subsample with complete Y, and so $W$ can be imputed in this subsample. This provides an example where restricting to a complete outcome variable will result in unbiased estimation of the target parameter via subsample-MI.

Figure S2B is the same as Figure S2A, except instead of a direct path from $Y$ to $R_{W}$, there is an unmeasured common cause $U$ of $Y$ and $R_{W}$. As before $W$ and $X$ cause $R_{Y}$, $X$ also causes $R_{W}$, $X$ is complete, and the set $\Phi$ consists of $\{W,Y\}$, so the m-backdoor criterion does not hold and MI in the whole sample may not be valid. Separating the incomplete variables $Z’$ into $P$ (response dependent on $Y$) and $Q$ (response independent of $Y$) again yields $P=W$ and $Q=Y$, i.e. imputing W in the subsample with complete values of $Y$. However, the check for independence between $W$ and all response indicators (the m-backdoor criterion) fails as $Y$ is a collider for $W$ and the unmeasured variable $U$, resulting in an open path between $W$ and $R_{W}$ when we condition on $Y$ (which is now a complete variable in this subsample and would be conditioned on in the imputation model for $W$). The data are therefore not z-MAR in the subsample with complete $Y$ and it may not be possible to unbiasedly estimate the target parameter using subsample-MI using any subsample of observed variables. The comparison between Figure S2A and S2B highlights the importance of temporality of variables. It is more likely that there is common cause of $Y$ and response for the earlier occurring variable $W$ than for $Y$ to directly cause response in $W$. It is therefore possible to subsample on observed values of the outcome but is unlikely to be feasible in practice due to collider bias. If instead the variable $U$ is measured and complete, then it can be included in the imputation model as an auxiliary variable to close the path between $W$ and $R_{W}$ conditional on $Y$, making the data z-MAR in the subsample with complete $Y$.

In Figure S2C the variable $X$ is additionally incomplete. $W$ causes $R_{X}$ and $R_{Y}$, and Y causes $R_{W}$. Following steps 1-3 of the algorithm, the set $\Phi$ consists of $X,W,Y$ and MI in the full sample may not be valid. We note that we cannot subsample on complete $W$, because $Y$ causes $R_{W}$. Instead, we could subsample on complete values for the variables $X$ and $Y$ - partitioning incomplete variables $Z’$ into $P=\{W\}$ and $Q=\{Y,X\}$. Subsampling on complete $Y$ and $X$ (i.e. restricting to those individuals with both $Y$ and $X$ fully observed), results in the data being z-MAR in the subsample as $W$ is no longer related to a response indicator for an incomplete variable, conditional on $X$ and $Y$ being complete in this subsample. This scenario shows that it is possible, and sometimes necessary, to subsample on observed values of multiple incomplete variables to unbiasedly estimate the target parameter via subsample-MI, though we note that the temporality issue described above in Figure S2B is still relevant here.

Finally, Supplementary Figure S3 is an example provided in our previous work exploring incomplete auxiliary variables ^20^. In this example $X$ causes $Y$ and $R_{Y}$, and an auxiliary variable $A$ causes $Y$ and its own missingness ($R_{A}$). In this example the target parameter is the unconditional regression coefficient of $Y$ on $X$. In our previous work we showed via simulation that the target parameter is unbiasedly estimated using CRA and MI excluding the auxiliary variable from the imputation model but not using MI including the auxiliary in the imputation model. Implementing the algorithm in the absence of $A$ gives $\Phi=\emptyset$ and hence MI excluding the auxiliary in the whole dataset is valid. Implementing the algorithm in the presence of the auxiliary gives $\Phi=\{Y,A\}$ and hence MI including the auxiliary in the whole dataset is not valid. Our algorithm steps 1-3 predict the previous simulation finding for both settings. We then investigate separating $Z’=\{Y,A\}$ into $P=\{A\}$ and $Q=\{Y\}$. Restricting to complete $Q$ (i.e. complete $Y$) simply gives the CRA sample and so subsample-MI in this example will not provide any efficiency gains beyond CRA. Separating $Z’=\{Y,A\}$ into $P=\{Y\}$ and $Q=\{A\}$ and restricting to complete $Q$ (i.e. complete A) will estimate the target parameter with bias because $R_{A}$ is dependent on $Y$, conditional on $X$ (we do not condition on $A$ here because it is not in the analysis model). Thus, in this example we would choose to use CRA to estimate the target parameter as 1) MI of the outcome in the whole dataset using no auxiliaries would also be unbiased but less precise than CRA ^21^, 2) MI of the outcome in the whole dataset including the auxiliary would be biased and 3) subsample-MI would either be biased ($Q=\{A\}$) or less precise than CRA ($Q=\{Y\})$.


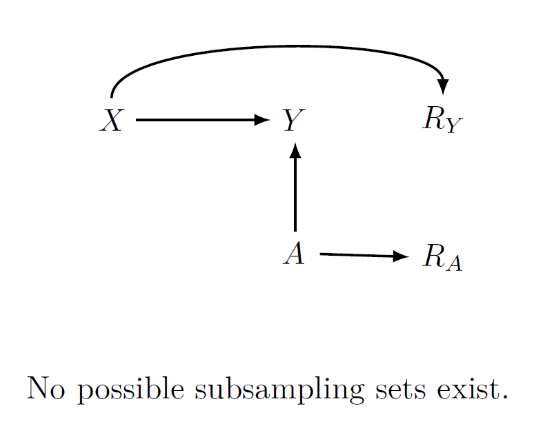


Figure S3: Additional example including an incomplete auxiliary variable.

# Algorithm applied to canonical missingness DAGs of Moreno-Betancur et al 2018 ^8^

- Analysis is Y | X, Z_1_, Z_2_, i.e. the target parameter is the regression coefficient for X in the regression of Y on X adjusted for Z_1_ and Z_2_.
- Z_1_ variables are fully observed.
- No auxiliary variables
- U and W are unmeasured


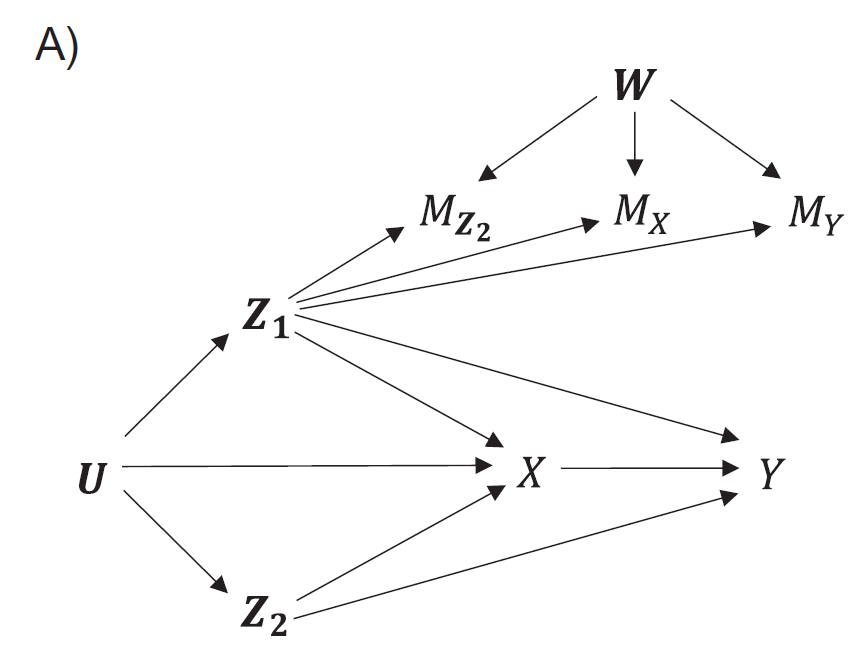


- Missingness in Y depends on Z_1_.
- Missingness in X depends on Z_1_.
- Missingness in Z_2_ depends on Z_1_.

Apply algorithm to full sample

Step 1) Y, X and Z_2_ are incomplete, Z_1_ is complete

Step 2) Φ = {}

Step 3) Φ is empty; exit algorithm. Impute Y, X and Z_2_ based on entire sample.

**Can apply standard MI to entire sample since the data are z-MAR given fully observed Z_1_.**


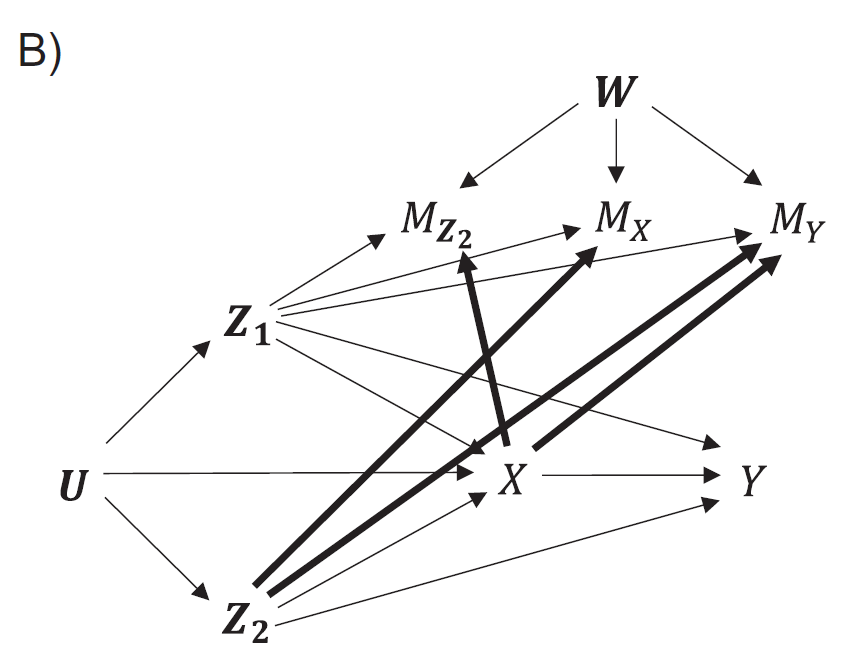


- Missingness in Y depends on X, Z_1_, Z_2_.
- Missingness in X depends on Z_1_, Z_2_.
- Missingness in Z_2_ depends on X, Z_1_.

Apply algorithm to full sample

Step 1) Y, X and Z_2_ are incomplete, Z_1_ is complete

Step 2) Φ =X, Z_2_, Y

Step 3) Φ is not empty; estimate from MI applied to the full dataset may be biased.

Apply algorithm to subsample $M^{X}$=0 and $M^{Z_{2}}$=0

Step 1, Modification 1) Q =X, Z_2_, P=Y

Step 1, Modification 2) Restrict to $M^{X}$=0 and $M^{Z_{2}}$=0

Step 2) Within this subsample, Φ is empty (i.e. all incomplete variables are d-separated from response indicators by complete variables).

Step 3) Estimate from MI of Y within the subsample $M^{X}$=0 and $M^{Z_{2}}$=0 will be unbiased.

- **In the full sample, the data are not z-MAR.**
- **Within subsample** $\boldsymbol{M}^{\boldsymbol{X}}\boldsymbol{=0,}\boldsymbol{M}^{\boldsymbol{Z}_{\boldsymbol{2}}}\boldsymbol{=0}$**, the data are z-MAR given fully observed variables.**
- **No smaller subsamples can be found where the estimate from MI will be unbiased.**


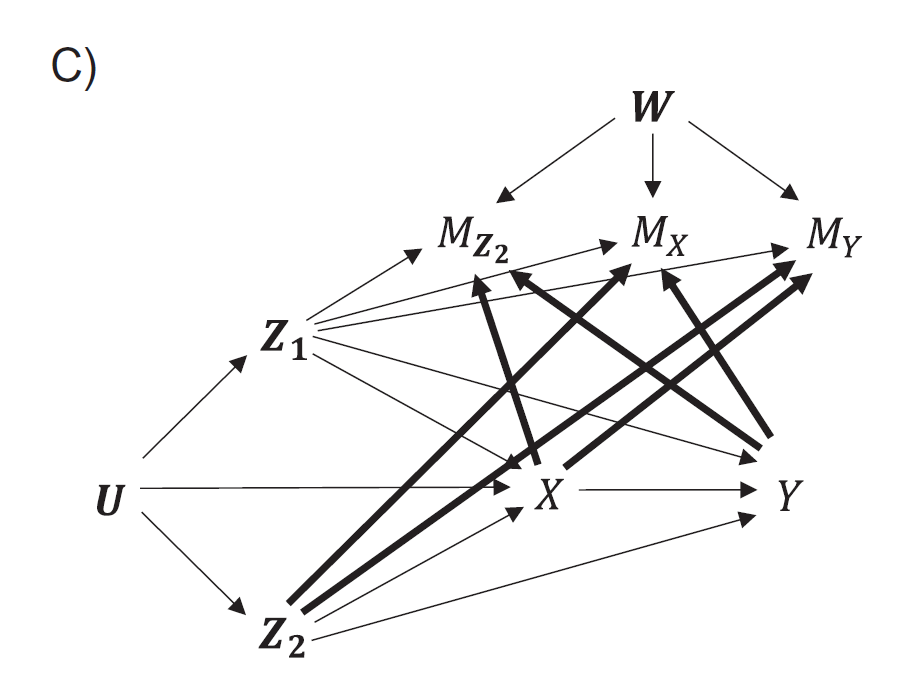


- Missingness in Y depends on X, Z_1_, Z_2_.
- Missingness in X depends on Y, Z_1_, Z_2_.
- Missingness in Z_2_ depends on X, Y, Z_1_.

Apply algorithm to full sample

Step 1) Y, X and Z_2_ are incomplete, Z_1_ is complete

Step 2) Φ =X, Y, Z2

Step 3) Φ is not empty; so the estimate from MI applied to the full dataset may be biased.

Apply algorithm to subsample $M^{Y}$=0

Step 1, Modification 1) Q=Y, P =X, Z_2_,

Step 1, Modification 2) Restrict to $M^{Y}$=0

Step 2) Within this subsample, Φ = {X, Z_2_}

Step 3) Φ is not empty, so the estimate from MI of X, Z_2_ within the subsample with complete Y ( $M^{Y}$=0) will be biased.

Applying algorithm to other partitions is not possible because Y causes response indicators for X and Z_2_.

- **In the full sample, the data are not z-MAR.**
- **Within subsample** $\boldsymbol{M}^{\boldsymbol{Y}}\boldsymbol{=0}$**, the data are not z-MAR for missing data pattern jointly missing X and Z_2_.**


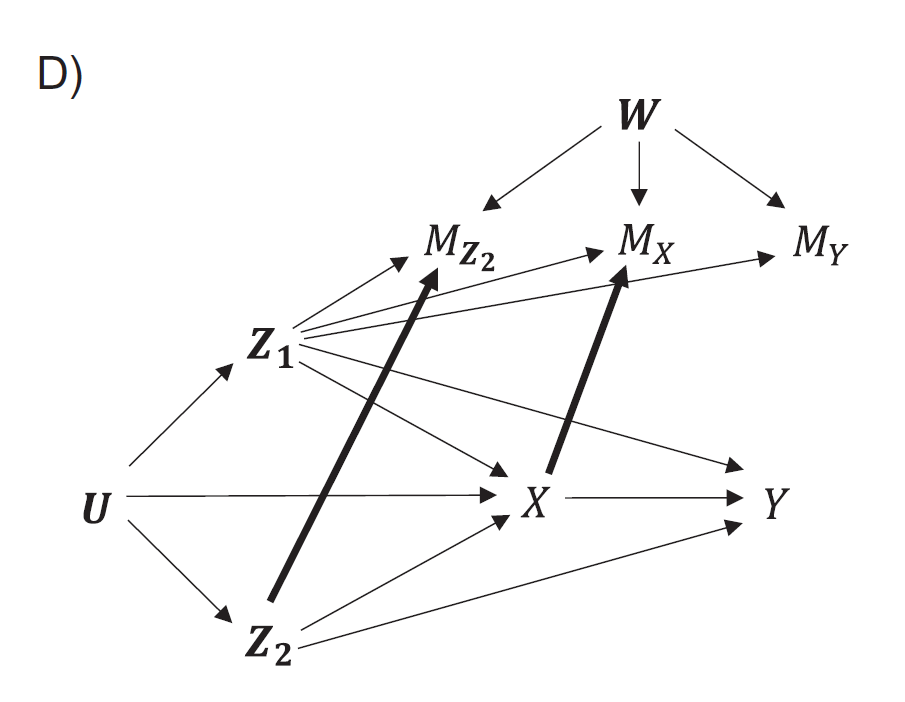


- Missingness in Y depends on Z_1_.
- Missingness in X depends on X, Z_1_.
- Missingness in Z_2_ depends on Z_1_, Z_2_.

Apply algorithm to full sample

Step 1) Y, X and Z_2_ are incomplete, Z_1_ is complete

Step 2) Φ =X, Z_2_, Y

Step 3) Φ is not empty; estimate from MI applied to the full dataset may be biased.

Apply algorithm to subsample $M^{X}$=0 and $M^{Z_{2}}$=0

Step 1, Modification 1) Q =X, Z_2_, P=Y

Step 1, Modification 2) Restrict to $M^{X}$=0 and $M^{Z_{2}}$=0

Step 2) Within this subsample, Φ is empty (i.e. all incomplete variables are d-separated from response indicators by complete variables).

Step 3) Estimate from MI of Y within the subsample $M^{X}$=0 and $M^{Z_{2}}$=0 will be unbiased.

- **In the full sample, the data are not z-MAR due to paths from incomplete variables X and Z2 to response indicators.**
- **Within subsample** $\boldsymbol{M}^{\boldsymbol{X}}\boldsymbol{=0,}\boldsymbol{M}^{\boldsymbol{Z}_{\boldsymbol{2}}}\boldsymbol{=0}$**, the data are z-MAR given fully observed variables.**
- **No smaller subsamples can be found where the estimate from MI will b unbiased.**


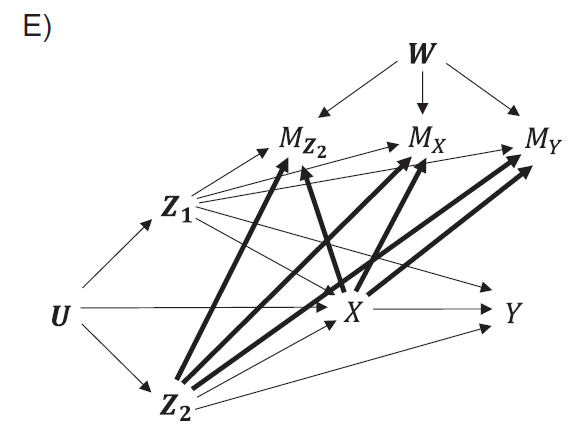


- Missingness in Y depends on Z_1_, X, and Z_2_.
- Missingness in X depends on X, Z_1_, Z_2_.
- Missingness in Z_2_ depends on X, Z_1_, Z_2_.

Apply algorithm to full sample

Step 1) Y, X and Z_2_ are incomplete, Z_1_ is complete

Step 2) Φ =X, Z_2_, Y

Step 3) Φ is not empty; estimate from MI applied to the full dataset may be biased.

Apply algorithm to subsample $M^{X}$=0 and $M^{Z_{2}}$=0

Step 1, Modification 1) Q =X, Z_2_, P=Y

Step 1, Modification 2) Restrict to $M^{X}$=0 and $M^{Z_{2}}$=0

Step 2) Within this subsample, Φ is empty (i.e. all incomplete variables are d-separated from response indicators by complete variables).

Step 3) Estimate from MI of Y within the subsample $M^{X}$=0 and $M^{Z_{2}}$=0 will be unbiased.

- **In the full sample, the data are not z-MAR due to paths from incomplete variables X and Z_2_ to response indicators. Within subsample** $\boldsymbol{M}^{\boldsymbol{X}}\boldsymbol{=0,}\boldsymbol{M}^{\boldsymbol{Z}_{\boldsymbol{2}}}\boldsymbol{=0}$**, the data are z-MAR given fully observed variables.**


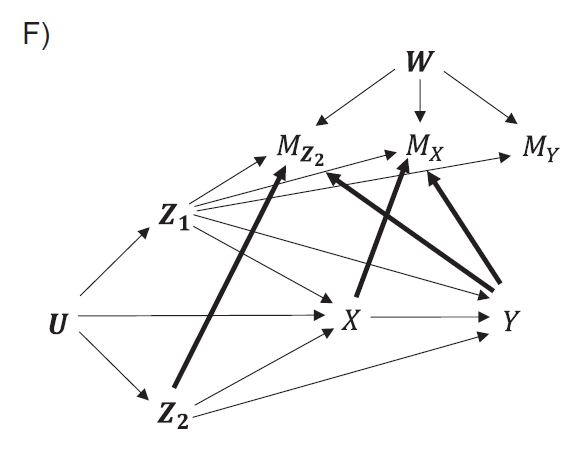


- Missingness in Y depends on Z_1_.
- Missingness in X depends on Y, X, Z_1_.
- Missingness in Z_2_ depends on Y, Z_1_, Z_2_.

Apply algorithm to full sample

Step 1) Y, X and Z_2_ are incomplete, Z_1_ is complete

Step 2) Φ =Y, X, Z_2_

Step 3) Φ is not empty; so, the estimate from MI applied to the full dataset may be biased.

Apply algorithm to subsample $M^{Y}$=0

Step 1, Modification 1) Q=Y, P =X, Z_2_,

Step 1, Modification 2) Restrict to $M^{Y}$=0

Step 2) Within this subsample, Φ = {X, Z_2,_}

Step 3) Φ is not empty, so the estimate from MI of X, Z_2_ within the subsample with complete Y ( $M^{Y}$=0) will be biased.

Applying the algorithm to other partitions is not possible because Y causes response indicators for X and Z_2_.

- **In the full sample, the data are not z-MAR due to paths from incomplete variables X, Z_2_ and Y to response indicators. Within subsample** $\boldsymbol{M}^{\boldsymbol{Y}}\boldsymbol{=0}$**, the data the data are not z-MAR due to paths from incomplete variables X and Z_2_ to response indicators.**


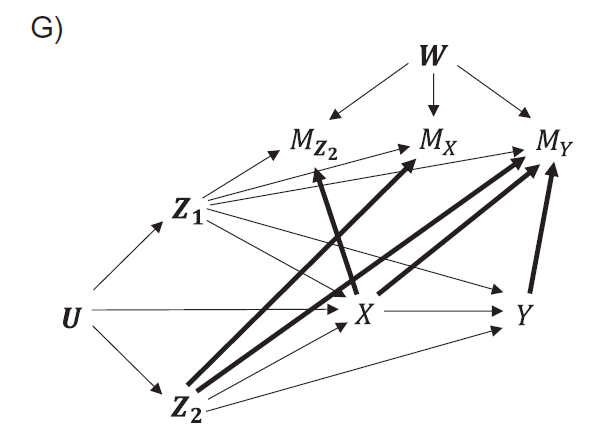


- Missingness in Y depends on Y, X, Z_1_, Z_2_.
- Missingness in X depends on Z_1_, Z_2_.
- Missingness in Z_2_ depends on X, Z_1_.

Missingness in Y depends on itself. There is no need to apply the full algorithm, as we know that Y cannot be imputed (as incomplete Y will always cause the data to not be z-MAR) and we cannot apply MI in the sample in which Y is complete (because Y causes inclusion in that subsample). Thus, MI applied to the full sample, or to any subsample, may estimate the target parameter with bias.


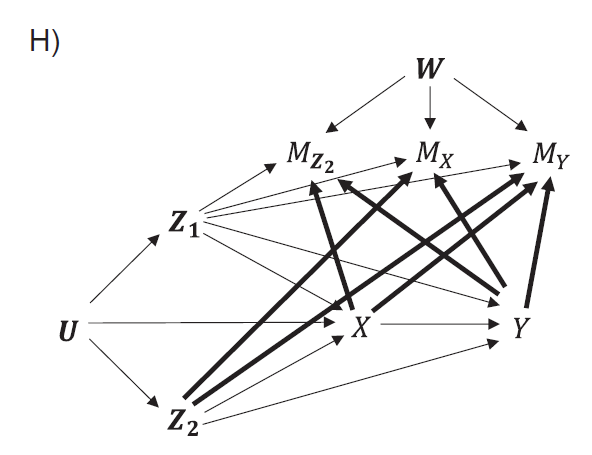


- Missingness in Y depends on Y, X, Z_1_, Z_2_.
- Missingness in X depends on Y, Z_1_, Z_2_.
- Missingness in Z_2_ depends on Y, X, Z_1_.

Missingness in Y depends on itself. There is no need to apply the full algorithm, as we know that Y cannot be imputed (as incomplete Y will always cause the data to not be z-MAR) and we cannot apply MI in the sample in which Y is complete (because Y causes inclusion in that subsample). Thus, MI applied to the full sample, or to any subsample, may estimate the target parameter with bias.

| 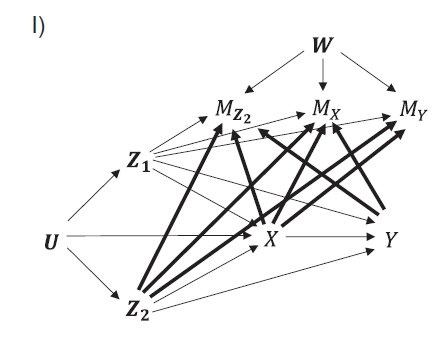 | Missingness in Y depends on X, Z_1_, Z_2_.  Missingness in X depends on Y, X, Z_1_, Z_2_.  Missingness in Z_2_ depends on Y, X, Z_1_, Z_2_ |
| --- | --- |

Apply algorithm to full sample

Step 1) Y, X and Z_2_ are incomplete, Z_1_ is complete

Step 2) Φ =Y, X, Z_2_

Step 3) Φ is not empty; so, the estimate from MI applied to the full dataset may be biased.

Apply algorithm to subsample $M^{Y}$=0

Step 1, Modification 1) Q=Y, P =X, Z_2_,

Step 1, Modification 2) Restrict to $M^{Y}$=0

Step 2) Within this subsample, Φ = {X, Z_2_}

Step 3) Φ is not empty, so the estimate from MI of X, Z_2_ within the subsample with complete Y ( $M^{Y}$=0) will be biased.

Applying algorithm to other partitions is not possible because Y causes response indicators for X and Z_2_.

| 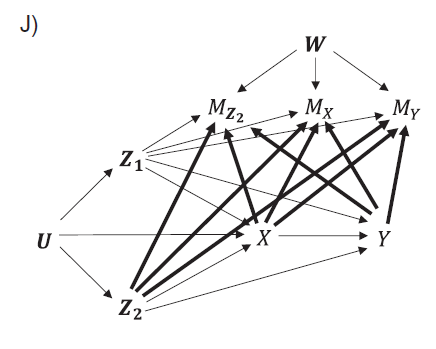 | Missingness in Y depends on Y, X, Z_1_, Z_2_.  Missingness in X depends on Y, X, Z_1_, Z_2_.  Missingness in Z_2_ depends on Y, X, Z_1_, Z_2_ |
| --- | --- |

Missingness in Y depends on itself. There is no need to apply the full algorithm, as we know that Y cannot be imputed (as incomplete Y will always cause the data to not be z-MAR) and we cannot apply MI in the sample in which Y is complete (because Y causes inclusion in that subsample). Thus, MI applied to the full sample, or to any subsample, may estimate the target parameter with bias.

# Figures showing application of algorithm to the motivating example


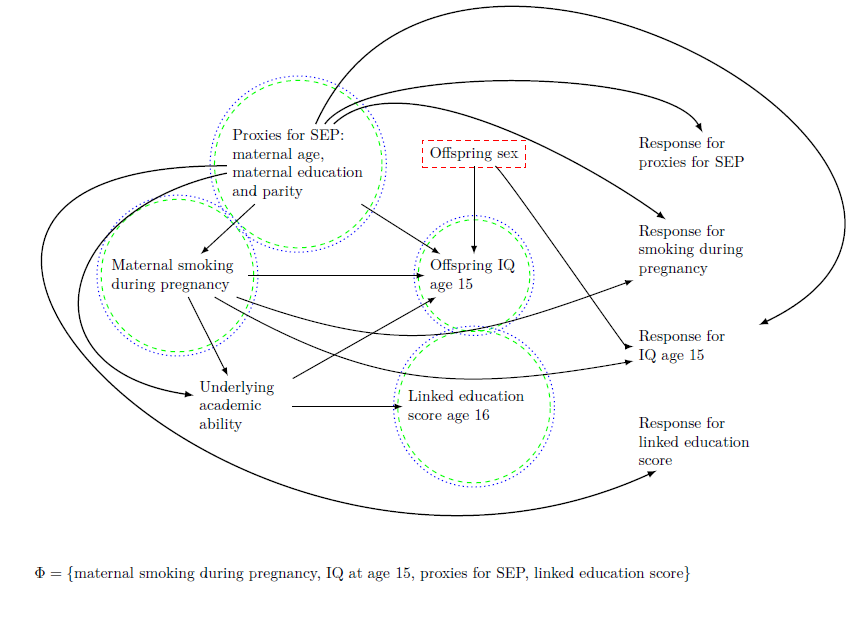


Figure S4: Application of the algorithm to the motivating example to establish whether the m-backdoor criterion holds in the whole sample. As $\Phi$ is not the empty set (there are variables with both a dotted blue and dashed green circle around them) the m-backdoor criterion does not hold and MI in the whole sample is not valid.


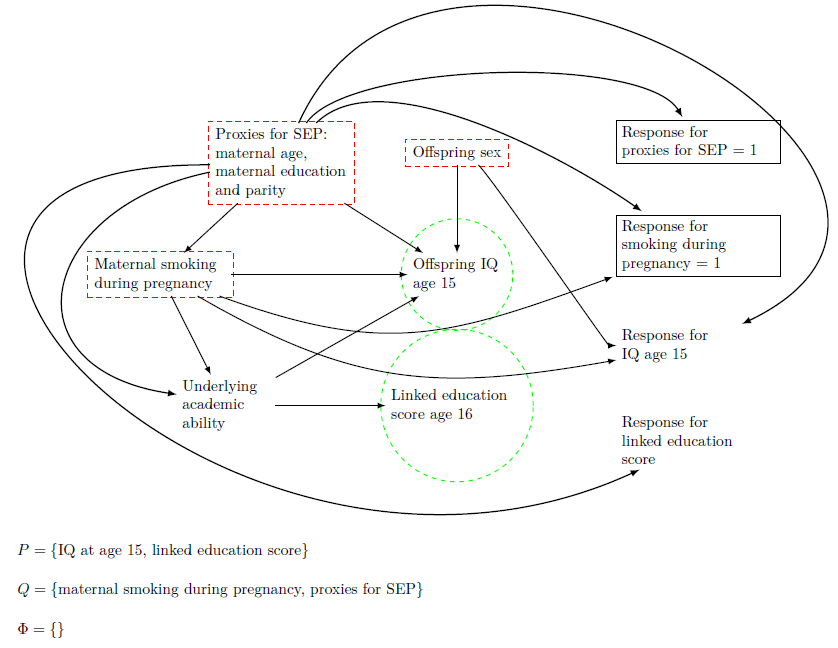


Figure S5: Application of the algorithm to the motivating example to establish whether the m-backdoor criterion holds in a subsample restricted to observed values for some variables. Following the modifications to step 1 and then reapplying steps 2 and 3 we set $Q$ = {maternal smoking during pregnancy, proxies for SEP} and $P$ = {IQ at age 15, linked education score}. Here the outcome variable is d-separated from all response indicators for variables in $Q$ conditional on complete variables and variables in $Q$. Note that we could not use $Q$ = {proxies for SEP} as there is still an open path from maternal smoking during pregnancy to its own response indicator. As $\Phi$ is the empty set (there are no variables with both a dotted blue and dashed green circle around them) the m-backdoor criterion holds and MI in sample with observed values of maternal smoking during pregnancy and SEP confounders is valid for estimating the conditional regression coefficient for the effect of maternal smoking in pregnancy on offspring IQ at age 15.

# Simulation study

We describe below the methods and results for a simulation study (using the ADEMP framework ^22^) exploring the scenarios described in Figures 1/3A, S1/3B, 4 and S2. All simulations were conducted in Stata 18.5MP. Code for the simulations can be found at <https://github.com/pmadleydowd/Subsample-MI>.

## Simulation study methods

### Aims

The simulation study aims to show the bias associated with complete records analysis, MI using all available data, and subsample-MI for the scenarios described in Figures 1/3A, S1/3B, 4 and S2 in the main text and earlier in the supplement.

### Data-generating mechanisms

Throughout the simulation studies, the target parameter of interest (regression coefficient for $Y$ on $X$, adjusted for $W$ where appropriate) has a true value of 0.15. The variables $U$, $W$, $X$ and $Y$ were simulated to have mean 0 and variance 0.5.

**Figure 1/3A**

We then simulated $X$, $Y$, probability of observed $X$ and probability of observed $Y$ as:

$$X\sim N\left( 1,0.5 \right)$$

$Y=0.15X+0.85+(\sqrt{{0.5}^{2}-{0.15}^{2})}\times\varepsilon$ where $\varepsilon\sim N\left( 0,1 \right)$

$$P\left( \text{observe }X \right)=0.5$$

$$P\left( \text{observe }Y \right)=0.9\text{ if }X<\text{median}\left( X \right), 0.1\text{ otherwise}$$

**Figure S1/3B**

We then simulated $X$, $Y$, probability of observed $X$ and probability of observed $Y$ as:

$$X\sim N\left( 1,0.5 \right)$$

$Y=0.15X+0.85+(\sqrt{{0.5}^{2}-{0.15}^{2})}\times\varepsilon$ where $\varepsilon\sim N\left( 0,1 \right)$

$$P\left( \text{observe }X \right)=0.9\text{ if} Y<\text{median}\left( Y \right), 0.1\text{ otherwise}$$

$$P\left( \text{observe }Y \right)=0.5$$

**Figure 4**

We simulated $W$, $X$, $Y$, and the probability of observing each of $W$, $X$ and $Y$ as:

$$W\sim N\left( 1,0.5 \right)$$

$X=\sqrt{0.5}W+(1-\sqrt{0.5})+(\sqrt{{0.5}^{2})}\times0.5\times\varepsilon$ where $\varepsilon\sim N\left( 0,1 \right)$

$Y=0.15X-0.5W+1.35+\left( \sqrt{0.25-\left( {0.15}^{2}+\left( \frac{{0.5}^{2}}{4} \right)-2\times0.15\times0.5\times\sqrt{0.5}/4 \right)} \right)\times\varepsilon$ where $\varepsilon\sim N\left( 0,1 \right)$

$$P\left( \text{observe }X \right)=0.9\text{ if} W<\text{median}\left( W \right), 0.1\text{ otherwise}$$

$$P\left( \text{observe }W \right)=0.9\text{ if} X<\text{median}\left( X \right), 0.1\text{ otherwise}$$

$$P\left( \text{observe }Y \right)=0.5$$

**Figure S2A**

We simulated $W$, $X$, $Y$ as for scenario 3, and the probability of observing $W$, $X$ and $Y$ as:

$$P\left( \text{observe }W \right)=0.9\text{ if} (X<\text{median}\left( X \right))\&(Y<\text{median}\left( Y \right)), 0.1\text{ if} (X>\text{median}\left( X \right))\&\left( Y>\text{median}\left( Y \right) \right), 0.5 \text{otherwise}$$

$$P\left( \text{observe }X \right)=1$$

$$P\left( \text{observe }Y \right)=0.9\text{ if} (X<\text{median}\left( X \right))\&(W<\text{median}\left( W \right)), 0.1\text{ if} (X>\text{median}\left( X \right))\&\left( W>\text{median}\left( W \right) \right), 0.5 \text{otherwise}$$

**Figure S2B**

We simulated $U$, $W$, $X$, $Y$, and the probability of observing each of $W$, $X$ and $Y$ as:

$$W\sim N\left( 1,0.5 \right)$$

$$U\sim N\left( 1,0.5 \right)$$

$X=\sqrt{0.5}W+\left( 1-\sqrt{0.5} \right)+\left( \sqrt{0.5} \right)\times0.5\times$ where $\varepsilon\sim N\left( 0,1 \right)$

$$Y=0.15X-0.5W+2-U\times\left( \sqrt{2\left( 0.25-\left( \frac{{0.15}^{2}}{4}+\left( \frac{{0.5}^{2}}{4} \right)-2\times0.15\times0.5\times\frac{\sqrt{0.5}}{4} \right) \right)} \right)+$$

$\left( 0.5\times\sqrt{2\left( 0.25-\left( \frac{{0.15}^{2}}{4}+\left( \frac{{0.5}^{2}}{4} \right)-2\times0.15\times0.5\times\sqrt{0.5}/4 \right) \right)} \right)\times\varepsilon$ where $\varepsilon\sim N\left( 0,1 \right)$

$$P\left( \text{observe }X \right)=1$$

$$P\left( \text{observe }W \right)=0.9\text{ if} (X<\text{median}\left( X \right))\&(U<\text{median}\left( U \right)), 0.1\text{ if} (X>\text{median}\left( X \right))\&\left( U>\text{median}\left( U \right) \right), 0.5 \text{otherwise}$$

$$P\left( \text{observe }Y \right)=0.9\text{ if} (X<\text{median}\left( X \right))\&(W<\text{median}\left( W \right)), 0.1\text{ if} (X>\text{median}\left( X \right))\&\left( W>\text{median}\left( W \right) \right), 0.5 \text{otherwise}$$

**Figure S2C**

We simulated $W$, $X$, $Y$, as for scenario 3, and the probability of observing $W$, $X$ and $Y$ as:

$$P\left( \text{observe }X \right)=0.8\text{ if} (W>\text{70th centile}\left( W \right), 0.4 \text{otherwise}$$

$$P\left( \text{observe }W \right)=0.9\text{ if} (Y<\text{median}\left( Y \right)), 0.1 \text{otherwise}$$

$$P\left( \text{observe }Y \right)=0.9\text{ if} (W<\text{median}\left( W \right), 0.1 \text{otherwise}$$

### Estimand/target of analysis

In each case, the estimand of interest was the coefficient for the linear regression of $Y$ on $X$ (unconditional for Figure 1/3A and S1/3B and conditional on $W$ for Figures 4 and S2).

### Methods to be evaluated

In each case, the complete records analysis and the MI using all available data were performed, along with the specified subsample-MI analyses.

### Performance measures

500 simulations were carried out for each example. We estimate 1) the average bias across simulations of the coefficient for the linear regression of Y on X (conditional on W where relevant) relative to the true value of 0.15, and 2) the empirical standard error of the bias across simulations.

## Simulation study results

Results for one set of parameters for Figures 1-S2C. In all cases true parameter value is 0.15.

| Scenario | Target parameter biased or unbiased using specified method as predicted by DAG/algorithm | | | Average bias in coefficient for regression of Y on X given W across 500 simulations (empirical SE of bias across 500 simulations) | | | |
| --- | --- | --- | --- | --- | --- | --- | --- |
|  | CRA | MI in whole sample | Subsample-MI | Bias in CRA | Bias in MI in whole sample | Subsample-MI | |
|  |  |  |  |  |  | “Complete” variable(s) | Bias |
| Figure 1 and 3A | Unbiased | Biased | Unbiased conditional on complete X or complete Y | 0.005 (0.086) | -0.034 (0.072) | Y | 0.003 (0.085) |
|  |  |  |  |  |  | X | 0.005 (0.087) |
| Figure S1 and 3B | Biased | Biased | Unbiased conditional on complete Y | -0.060 (0.052) | -0.039 (0.073) | Y | -0.002 (0.085) |
|  |  |  |  |  |  | X | -0.059 (0.052) |
| Figure 4A, B and C | Unbiased | Biased | Unbiased conditional on complete X, complete W, or complete X and W | 0.003 (0.118) | 0.073 (0.100) | X | -0.002 (0.090) |
|  |  |  |  |  |  | W | 0.003 (0.119) |
|  |  |  |  |  |  | X and W | 0.003 (0.112) |
| Figure S2A | Biased | Biased | Unbiased conditional on complete Y | -0.077 (0.083) | -0.022 (0.076) | Y | 0.000 (0.069) |
|  |  |  |  |  |  | W | -0.079 (0.084) |
| Figure S2B | Biased | Biased | Biased | 0.045 (0.082) | 0.063 (0.072) | Y | 0.018 (0.073) |
|  |  |  |  |  |  | W | 0.046 (0.084) |
| Figure S2C | Biased | Biased | Unbiased conditional on complete X and Y | -0.047 (0.005) | -0.402 (0.005) | Y | 0.018 (0.005) |
|  |  |  |  |  |  | W | -0.049 (0.005) |
|  |  |  |  |  |  | X and Y | 0.004 (0.006) |

CRA = Complete records analysis; MI = Multiple imputation; SE = Standard error

# Supplementary references

1. Zahid FM, Faisal S, Heumann C. Multiple imputation with compatibility for high-dimensional data. *PLoS One* 2021;16(7):e0254112. doi: 10.1371/journal.pone.0254112 [published Online First: 20210708]

2. Curnow E, Carpenter JR, Heron JE, et al. Multiple imputation of missing data under missing at random: compatible imputation models are not sufficient to avoid bias if they are mis-specified. *J Clin Epidemiol* 2023;160:100-09. doi: 10.1016/j.jclinepi.2023.06.011 [published Online First: 2023/06/22]

3. Hernan MA, Monge S. Selection bias due to conditioning on a collider. *BMJ* 2023;381:1135. doi: 10.1136/bmj.p1135 [published Online First: 20230607]

4. Hernán M, Robins J. Causal Inference: What If. November 10, 2019 ed. Boca Raton: Chapman & Hall/CRC 2020.

5. Pearl J. Causality : models, reasoning, and inference. Cambridge, U.K. ; New York: Cambridge University Press 2000.

6. Pearl J. Causal diagrams for empirical research (with Discussions). Probabilistic and causal inference: The works of Judea Pearl2022:255-316.

7. Pearl J. d-separation without tears (Chapter 1 of Causality, pp. 16-18) bayes.cs.ucla.edu [Available from: <https://bayes.cs.ucla.edu/BOOK-2K/d-sep.html> accessed 23 June 2025.

8. Moreno-Betancur M, Lee KJ, Leacy FP, et al. Canonical Causal Diagrams to Guide the Treatment of Missing Data in Epidemiologic Studies. *Am J Epidemiol* 2018;187(12):2705-15. doi: 10.1093/aje/kwy173 [published Online First: 2018/08/21]

9. Moreno-Betancur M, Lee KJ, Leacy FP, et al. Correction to: “Canonical causal diagrams to guide the treatment of missing data in epidemiologic studies”. *American Journal of Epidemiology* 2025 doi: 10.1093/aje/kwae406

10. Carpenter J, Kenward M. Multiple imputation and its application. 1 ed: John Wiley & Sons 2012.

11. Mathur MB, Shpitser I. Imputation without nightMARs: Graphical criteria for valid imputation of missing data. *Preprint retrieved from* [*https://osfio/preprints/osf/zqne9_v1*](https://osfio/preprints/osf/zqne9_v1) 2024 doi: <https://doi.org/10.31219/osf.io/zqne9> [published Online First: 30 July 2024]

12. Doretti M, Geneletti S, Stanghellini E. Missing Data: A Unified Taxonomy Guided by Conditional Independence. *International Statistical Review* 2018;86(2):189-204. doi: <https://doi.org/10.1111/insr.12242>

13. Rubin DB. Inference and Missing Data. *Biometrika* 1976;63(3):581-90. doi: DOI 10.1093/biomet/63.3.581

14. Rubin DB. Multiple imputation for nonresponse in surveys: New York: Wiley, 1987.

15. Seaman S, Galati J, Jackson D, et al. What Is Meant by “Missing at Random”? *Statistical Science* 2013;28(2):257-68, 12.

16. Mohan K, Pearl J. Graphical models for processing missing data. *J Am Stat Assoc* 2021:1-42.

17. Thoemmes F, Mohan K. Graphical Representation of Missing Data Problems. *Structural Equation Modeling: A Multidisciplinary Journal* 2015;22(4):631-42. doi: 10.1080/10705511.2014.937378

18. Little RJ, Zhang N. Subsample ignorable likelihood for regression analysis with missing data. *Journal of the Royal Statistical Society: Series C (Applied Statistics)* 2011;60(4):591-605.

19. Allison P. The Peculiarities of Missing at Random 2017 [22 July 2022]. Available from: <https://statisticalhorizons.com/missing-at-random/>.

20. Madley-Dowd P, Curnow E, Hughes RA, et al. Analyses Using Multiple Imputation Need to Consider Missing Data in Auxiliary Variables. *Am J Epidemiol* 2024 doi: 10.1093/aje/kwae306 [published Online First: 20240827]

21. Hughes RA, Heron J, Sterne JAC, et al. Accounting for missing data in statistical analyses: multiple imputation is not always the answer. *Int J Epidemiol* 2019;48(4):1294-304. doi: 10.1093/ije/dyz032 [published Online First: 2019/03/18]

22. Morris TP, White IR, Crowther MJ. Using simulation studies to evaluate statistical methods. *Stat Med* 2019;38(11):2074-102. doi: 10.1002/sim.8086 [published Online First: 20190116]
